# A Revolutionary Blueprint for Mitigation of Hypertension via Nanoemulsion

**DOI:** 10.1155/2022/4109874

**Published:** 2022-04-14

**Authors:** Girish Kumar, Tarun Virmani, Kamla Pathak, Abdulsalam Alhalmi

**Affiliations:** ^1^School of Pharmaceutical Sciences, MVN University, Haryana 121105, India; ^2^Uttar Pradesh University of Medical Sciences, Etawah, Uttar Pradesh 206001, India; ^3^Department of Pharmaceutics, College of Pharmacy, Aden University, Aden, Yemen

## Abstract

Hypertension is one of the most important causes of mortality, affecting the health status of the patient. At the same time, hypertension causes a huge health and economic burden on the whole world. The incidence and prevalence of hypertension are rising even among young people in both urban as well as rural communities. Although various conventional therapeutic moieties are available for the management of hypertension, they have serious flaws such as hepatic metabolism, reduced dose frequency, poor aqueous solubility, reduced bioavailability, and increased adverse effects, making the drug therapy ineffective. Therefore, it is required to design a novel drug delivery system having the capability to solve the constraints associated with conventional treatment of hypertension. Nanotechnology is a new way of using and manipulating the matter at the molecular level, whose functional organization is measured in nanometers. The applications of nanotechnology in the field of medicine provide an alternative and novel direction for the treatment of cardiovascular diseases and show excellent performance in the field of targeted drug therapy. Various nanotechnologies based drug delivery systems, such as solid lipid nanoparticles, nanosuspension, nanoemulsion, liposome, self-emulsifying systems, and polymeric nanoparticles, are available. Among them, nanoemulsion has provided a niche to supplement currently available therapeutic choices due to numerous benefits like stability, ease of preparation, enhanced drug absorption, reduced hepatic metabolism, increased dose frequency, enhanced bioavailability, and encapsulation of hydrophilic as well as hydrophobic drugs. This present review provides an in-depth idea about progression in treatment of hypertension, constraints for antihypertensive drug therapy, need of nanoemulsions to overcome these constraints, comparative analysis of nanoemulsions over other nanostructure drug delivery systems, pharmacodynamics studies of nanoemulsions for treatment of hypertension, recent patents for drug-loaded nanoemulsions meant for hypertension, and marketed formulations of nanoemulsions for hypertension.

## 1. Introduction

Hypertension is a severe medical condition that raises the threat of brain, heart, and kidney disorders dramatically specified by the perpetual high pressure in the blood vessels [1]. The systolic BP (the pressure exerted by the arterial walls upon the contraction of the heart) to the diastolic BP (the pressure exerted on the arterial walls upon the relaxation of the heart) ratio is a typical way to express blood pressure [2]. It is a serious public health concern and one of the leading causes of death globally. According to World Health Organization, approximately 1.3 billion people globally are affected by hypertension, with the majority (two-thirds) residing in low- and middle-income nations. In a survey conducted in the year 2015, it was shown that one in four women and one in five men are suffering from the problem of hypertension. It was estimated that only about one in every five patients with hypertension has their condition under control, and about 9 million deaths globally are attributed to hypertension globally. The worldwide noncommunicable disease target is to reduce the pervasiveness of hypertension by 25% between 2010 and 2025 [3]. A variety of conventional therapeutic agents acting through various mechanisms are available for the treatment of hypertension but suffers from various constraints like poor aqueous solubility, reduced bioavailability, hepatic metabolism, dose frequency, lack of organ targeting, and higher adverse effects, which can be defeated by the development of advanced systems for drug delivery [4, 5]. Advanced drug delivery systems involve the development of a nanotechnological technique, which is a rapidly developing sophisticated scientific field that encompasses a wide range of disciplines such as chemistry, physics, and biology, as well as unique nanodimension structures with therapeutic applications in pharmacology and the biomedical field [6–8]. Many researchers and scientists are interested in the development and standardization of nanoscale drug delivery systems for a variety of reasons. The nanodimension has a variety of features, including optical, magnetic, and structural surface area ratios, making it a fascinating topic of research in every aspect. It is used as a nanocarrier and a nanoadsorbent and functions as a nanocarrier of therapeutic agents, proteins, or probes, especially because the surface area of nanoscale therapeutics or devices is high [9–11]. These nanoscale approaches include solid lipid nanoparticles, nanoemulsion, nanosuspension, nanoparticles, liposomes, and self-emulsifying systems, among nanoemulsion seems to be a promising and exciting approach to address the constraints of conventional treatment employed for hypertension. Nanoemulsions are ideal drug delivery carriers as they have the potential to dissolve a large amount of lipophilic drugs, have higher compatibility, ability to shield drugs from hydrolysis and enzymatic degradation, and highly effective transportation system because of their extreme micro droplet size with large surface area. They are superior to microemulsions as they avoid the problems associated with microemulsions like coalescence, flocculation, or inherent creaming. Due to various advantages, nanoemulsion drug delivery systems are used in a variety of dosage forms (creams, sprays, foams, solutions, etc.) and result in widespread adoption of them in the pharmaceutical business [12–17]. The current article considers the constraints associated with conventional antihypertensive therapeutics, as well as the significance of the oral nanoemulsion drug delivery method in overcoming the constraints and improving hypertension treatment.

## 2. Hypertension

**Figure 1:**
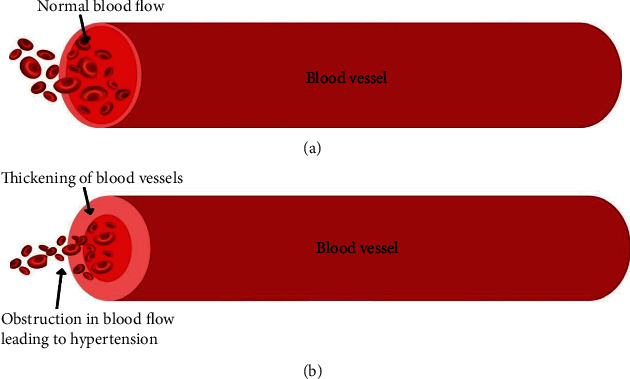
(a) Normal blood flow through a blood vessel and (b) obstruction in blood flow through a blood vessel leading to hypertension.

**Figure 2:**
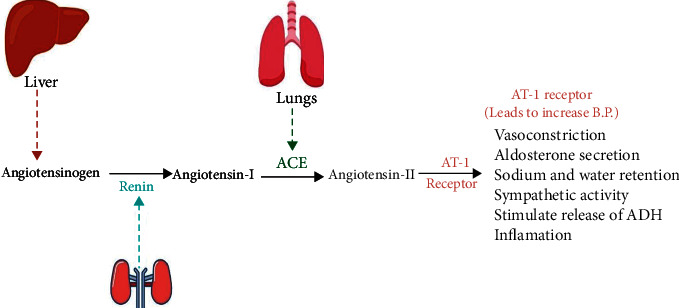
A schematic representation of various processes involved in increasing blood pressure.

Hypertension is one of the most important public health issues, widely acknowledged as the leading cause of global illness burden. It is a lifestyle illness that can be effectively treated by combining a healthy diet with frequent physical activity and maintaining a healthy weight [18]. It is a silent killer because no symptoms are visible in the early stages until a serious medical crisis such as a heart attack, stroke, or chronic renal failure occurs [19]. Because the majority of people are unaware of high blood pressure, the only way to detect it is through measurement. Although the majority of people suffering from hypertension are asymptomatic, some suffer from vertigo, headache, vision alteration, or fainting episodes [20, 21]. A combination of various factors persuading to hypertension and these factors fluctuate from country to country, and even within a country, there are differences between urban and rural communities [22]. When compared to their rural counterparts, city dwellers are more susceptible to certain ailments. According to the National Family Health Survey, India, the prevalence of hypertension was 10.5% in Uttar Pradesh's metropolitan areas and the prevalence of the same issue was 8.3% in rural areas, indicating the higher prevalence of hypertension in urban areas than in rural areas. Quick urbanization, an ageing population, mechanization, changing lifestyles, and dietary changes all contribute to a web of risk factors that entangles people leading to hypertension [20]. The vessels transport blood from the heart to all regions of the body. The heart pumps blood into the veins every time it beats. The force of blood pushing against the walls of blood vessels (arteries) as it is pumped by the heart causes blood pressure [23, 24]. The higher the pressure, the more difficult it is for the heart to pump causing hypertension as depicted using Figures 1(a) and 1(b), and the mechanism responsible for an increase in blood pressure causing hypertension is represented by Figure 2. The normal blood pressure is 120 mmHg (systolic pressure) and 80 mmHg (diastolic pressure), and beyond it, the condition of hypertension raised [25]. The management of hypertension is primarily linked to a reduction in modifiable risk factors and treatment [18].

### 2.1. Progression in Drug Treatment for Hypertension

**Figure 3:**
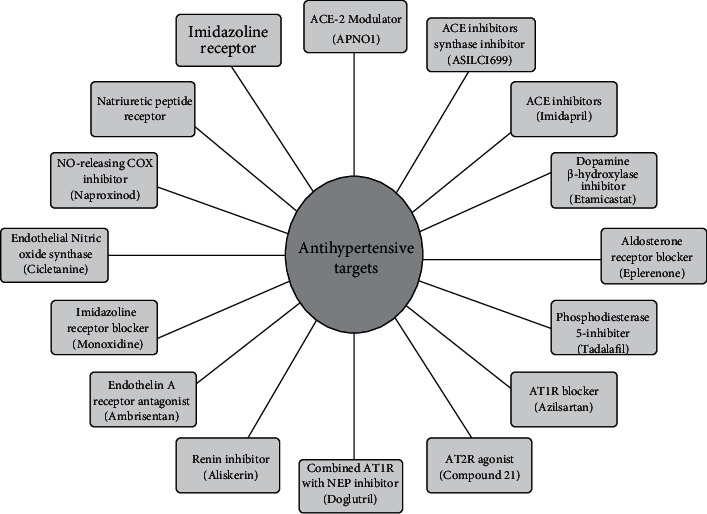
Novel targets for antihypertensive drugs.

Various drug molecules having different mechanisms are available for the treatment of hypertension. Pentaquine was the first drug molecule produced to treat hypertension in 1946; however, it had several side effects and had limited therapeutic efficacy. After this, hexamethonium was introduced in the early 1950s, and while it was effective, it was inconvenient to use [4]. When Veratrum was introduced, it was highly toxic, along with the quick onset of action [26]. Hydralazine was developed immediately after the negative effects of ganglionic blockers, and it is now rarely recommended. Because of its negative effects, such as depression and impotency, reserpine, the most successful medicine developed at the time, was also abandoned [27]. The contemporary age of hypertension therapy began in 1960 with breakthrough medications such as diuretics and *β*-blockers, which are now frequently prescribed. Drugs blocking calcium channels, inhibiting angiotensin-converting enzymes, and blocking angiotensin were originally developed in the 1990s and are now utilized as first-line therapy, either alone or in combination. The development of various newer drugs for hypertension has been aided by a rigorous consideration of the renin angiotensinogen aldosterone system. There has been significant progress in the development of innovative treatments, one of which has a target that is also related to the renin angiotensinogen aldosterone system [28, 29]. The unique targets that have opened up new avenues for the effective development of antihypertensive now in the preclinical and clinical stages of development are depicted in Figure 3.

### 2.2. Constraints Associated with Antihypertensive Drugs

In general, a drug's solubility and its permeability are critical parameters contributing to the oral absorption of the drugs. For a drug to exhibit higher oral absorption, both its solubility and its permeability must be higher. The drugs belonging to BCS class II exhibit poor aqueous solubility, contributing to the low bioavailability of the drugs [30, 31]. The majority of the antihypertensive drug molecules have poor aqueous solubility, leading to low bioavailability [13, 32]. Various antihypertensive drugs having poor bioavailability are summarized in Table 1, showing the class of drug, its aqueous solubility, permeability, and bioavailability, illustrating that poor aqueous solubility acts as the main constraint in oral absorption of the drugs attributing to low bioavailability.

### 2.3. Drug Delivery Systems for Antihypertensive Drugs

Drug delivery systems can be characterized as techniques for delivering therapeutic substances into the body [50]. In ancient times, drug delivery to treat hypertension was accomplished by grinding medicinal plants, leaves, or roots and inhaling the smoke of burning medicinal herbs. But these rudimentary techniques of drug delivery lacked a fundamental requirement in drug delivery: consistency and uniformity [51]. Hence, in the last eighteen and early nineteenth centuries, this resulted in the invention of various drug delivery systems for hypertension, which include pills, tablets, capsules, emulsions, suspensions, troches, lozenges, syrups, and various other systems, collectively known as conventional drug delivery systems [52]. These conventional drug delivery systems imposed various drawbacks: reduced bioavailability, lack of site-specificity, higher adverse effects, hepatic metabolism, a requirement for a large dose, fluctuation in steady-state concentration, and additionally the poor aqueous solubility of 90% of newly developed therapeutic moieties, prompting modification in conventional drug delivery systems and the invention of nanotechnology-based techniques [53, 54]. Nanotechnology-based techniques include liposomes, lipid-based nanoparticles, nanoemulsion, polymeric nanoparticles, dendrimers, and polymeric micelles, but nanoemulsion appears to be an exciting and promising drug delivery system to target the drugs in treatment of hypertension [55–57].

## 3. Nanoemulsion as Drug Delivery System

**Figure 4:**
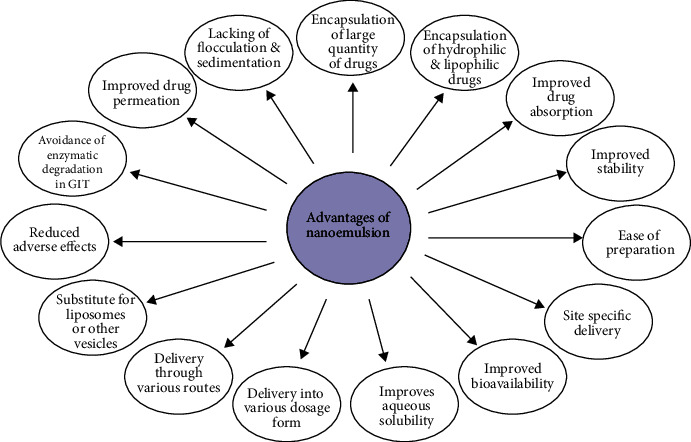
Various advantages of nanoemulsion as a drug delivery system.

**Figure 5:**
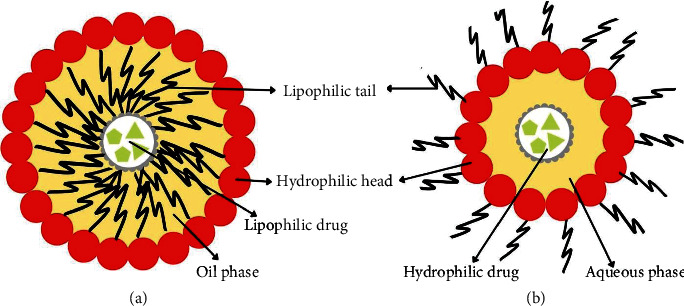
(a) Encapsulation of lipophilic drug in oil-in-water nanoemulsion and (b) encapsulation of hydrophilic drug in water-in-oil nanoemulsion.

Nanoemulsion may be defined as an isotropically clear, thermodynamically unstable colloidal dispersion made up of two immiscible phases along with surfactants and cosurfactants to produce a single phase [58]. Every droplet of nanoemulsion exhibits a diameter of 10 to 200 nm which provides various benefits over conventional as well as other modern approaches like an encapsulation of large quantity of the drug, encapsulation of hydrophilic as well as lipophilic drugs, enhanced drug absorption owing to reduced particle size, improved stability, ease of manufacturing, targeting to particular organ or tissue, improved bioavailability, improved aqueous solubility, delivery into several dosage forms, delivery into the body through various routes, substitute for liposomes or another vesicle, improved drug efficacy leading to reduced adverse effects, delivery of peptides prone to enzymatic degradation in GIT, improved drug permeation through the skin, and lack of limitations of macro emulsions like flocculation, creaming, and sedimentation, as represented by Figure 4 [59–64]. Depending on the presence of a disperse phase, the nanoemulsion can be classified into three categories: oil in water (O/W), water in oil (W/O), and multiple emulsion (W/O/W and O/W/O) [65, 66].  Figure 5 depicts the structure and composition of water in oil (W/O) and oil in water (O/W) nanoemulsions, with hydrophilic drugs enclosed within the W/O nanoemulsion and hydrophobic medications encapsulated within the O/W nanoemulsion, resulting in increased bioavailability [67]. The nanoemulsion is composed of the lipid/oil phase, surfactant, and cosurfactant summarized in Table 2 [68, 69].

### 3.1. Nanoemulsion as Therapeutics in Hypertension

**Figure 6:**
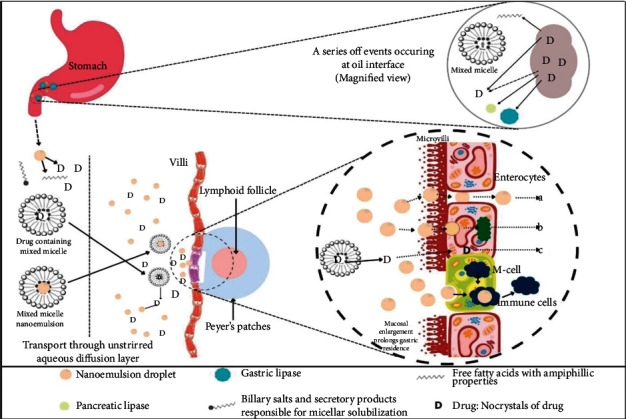
An understanding of the steps that occur within GIT that leads to the absorption of nanoemulsion. (a) The immediate absorption of the drug through lipid solubilization and partitioning processes dictates the lymphatic entrance. (b) Droplets could use paracellular or transcellular channels, M-cells, or mucosal entanglement for the uptake process of the drug. (c) Droplets may be converted into apolipoproteins and directed towards lymphatic drainage once within the absorptive cell.

It is reported that the majority of the antihypertensive drugs have low aqueous solubility, which is attributed to the low bioavailability of the drugs [70]. The advancement in drug delivery systems for antihypertensive drugs is nanotechnology, which will be either nanoparticles or nanoemulsion. The mainstay of the nanoemulsion approach in the management of hypertension is mainly achieved using the oral administration of antihypertensive drugs. Scientists and researchers will be more interested in formulations based on the nanoscale level in the management of hypertension [71, 72]. Since hypertension is associated with various consequences, researchers and formulation scientists are interested in using nanotechnology-based drug delivery systems for consequences associated with hypertension [73]. The administration of nanoemulsion through the oral route is most convenient to deliver the drug than other routes due to various benefits like self-administration, ease to administration, dose accuracy, patient compliance, and cost-effectiveness; primarily, oral administration is preferred [74–76]. In earlier times, various scientists have reported considerable improvement in the oral bioavailability of antihypertensive drugs having poor aqueous solubility and high hydrophobicity using the nanoemulsion technology summarized in Table 3. On administration of poorly aqueous soluble drugs using nanoemulsion, significant improvements in pharmacokinetic parameters (Cmax and AUC) were reported, demonstrating the pharmacokinetic benefit of the nanoemulsion over conventional technology [77–79]. Upon oral ingestion, the nanoemulsions enter the gastrointestinal system (GI tract) and are exposed to a variety of environmental variables [80]. In response, gastric lipase is secreted in the gastrointestinal system due to stimulation of the lipid sensing mechanism, enabling the fractional digestion of the lipid layer of nanoemulsion followed by the yielding of simpler diglycerides, monoglycerides, and free fatty acids [81, 82]. The lipase activity is accelerated by the nanoemulsion droplet's small size. The digestion of the lipid component of the nanoemulsion liberates the drugs, followed by nanoprecipitation [83]. In some cases, the drug will simply partition out of the lipid component into the surrounding aquatic environment. Lipids and lipid digested products in the gastrointestinal tract promote bile production and slow down gastrointestinal motility [84]. Bile components act as endogenous surfactants and may create colloidal structures termed “mixed micelles,” which promote nanoemulsion solubilization. Thus, bile and preexistent mixed micelles enable the solubilization of freed drug even more and transport it through the unstirred aqueous diffusion layer for absorption [82]. Paracellular or transcellular channels along with M-cells in Peyer's patches also provide intact absorption of the drugs through the oral route due to endocytosis followed by drug transport into intraepithelial spaces as depicted in Figure 6. Moreover, collisional absorption also happens implying the inadvertent impact absorption of nanoemulsion droplets [85]. Upon absorption, nanoemulsion may enter the systemic circulation through the hepatic portal vein or be interfaced into the perforated lymphatic endothelium [86]. Drugs absorbed through the lymphatic route are delivered directly into the blood circulation, bypassing the hepatic metabolism of the drugs [87]. Thus, various mechanisms interact to provide improvement in the oral bioavailability of drugs having poor aqueous solubility and low bioavailability when administered in the form of nanoemulsion.

### 3.2. Comparative Analysis of Nanoemulsion versus Another Nanostructured Delivery System

**Figure 7:**
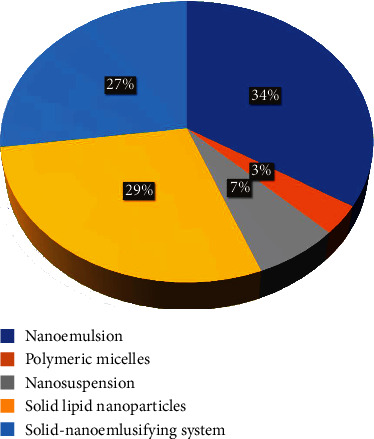
Pie chart depicted the segment of various novel drug delivery systems for delivering antihypertensive drugs.

This analysis was carried out by studying research publications published between 2016 and 2021 in the Science Direct, Pubmed, Springer, Taylor & Francis, Google Scholar, and EBSCO databases on nanostructured drug delivery systems to deliver antihypertensive drugs for the management of hypertension. Nanoemulsion for hypertension, novel drug delivery system for hypertension, advanced drug delivery system, solid lipid nanoparticles for antihypertensive drugs, polymeric micelles for hypertension, nanosuspension to deliver antihypertensive, self-nanoemulsifying drug delivery system for antihypertensive, and advanced drug delivery system for hypertension were the keywords to study the research publications. Only articles written in English were studied. Articles with possibly relevant titles were then evaluated based on their abstracts, with irrelevant articles being eliminated. After that, all of the publications with potentially relevant abstracts were thoroughly examined. In the meantime, the ones that were not relevant were eliminated. According to an exhaustive study of research publications from 2016 to 2021, nanoemulsion has captured a large market segment in comparison to other nanostructured drug delivery systems for delivering antihypertensive drugs.  Figure 7 using a pie chart depicted that nanoemulsion (34%) has a presiding role superseded by solid lipid nanoparticles (29%), self-nanoemulsifying drug delivery systems (27%), nanosuspension (7%), and polymeric micelles (1%) in descending order to deliver the antihypertensive drugs.

### 3.3. Pharmacodynamic Studies of Nanoemulsion Delivering Antihypertensive Drugs

According to a published research by Nada et al., it was found that unilateral ureteral obstruction rats that were given red ginger nanoemulsion had a substantial reduction in systolic blood pressure from 142 ± 1 mmHg to 107 ± 6 mmHg and diastolic blood pressure from 106 ± 1 mmHg to 84 ± 4 mmHg. Additionally, red ginger nanoemulsion therapy resulted in a 10.80% reduction in the level of ACE [88]. In a research study by Mundada et al., it was found that nisoldipine nanoemulsion had a significant reduction in systolic blood pressure from 187.31 ± 18.02 mmHg to 139.68 ± 13.12 mmHg and diastolic blood pressure from 122.36 ± 12.01 mmHg to 101.84 ± 10.28 mmHg [89]. In a research study by Gorain et al., it was observed that systolic blood pressure (115.71 ± 4.800 mmHg) was reduced after 1 hour of administration of Olmesartan nanoemulsion and was controlled up to 12 hours after administration (120.12 ± 5.724 mmHg). The considerable reduction in blood pressure persisted until the 14th day of treatment (108.96 ± 3.24 mmHg at 1 hour and 114.72 ± 3.74 mmHg at 12 hours after administration) until it reached the normal systolic blood pressure [94]. In a study by Chhabra et al., the uptake of amlodipine besilate from the nanoemulsion formulation was higher in almost every organ, especially in the heart than suspension of amlodipine besilate, confirming the targeting activity of nanoemulsion. Percentage uptake of the drug was found to be 11.8 and 13.6 folds greater in the heart at 0.5 hours and 1.0 hours, respectively, from nanoemulsion of amlodipine besilate than suspension of the drug [98].

### 3.4. Patents for Antihypertensive Nanoemulsion

A patent is the government's formal right to grant an inventor the sole right to create, sell, or use a product for a specific amount of time [103]. Several patents for nanoemulsion formulations have been issued, demonstrating the widespread acceptance of nanoemulsion formulations [104]. A patent (CN105997873A) was awarded to Zhang Hongli for the preparation of oil-in-water type nanoemulsion containing terazosin as an antihypertensive drug, aiming at prolonging the half-life of the drug, reducing dose frequency, and improving antihypertensive activity of the drug. The patent discloses oil-in-water type nanoemulsion composed of terazosin 1-15%, surfactant 15%-40%, cosurfactant 0-20%, and the remaining amount of distilled water. Ouyang Wuqing et al. granted a patent (CN102698245A) for the preparation of an antihypertensive drug of quinapril hydrochloride and rose oil nanoemulsion aiming at prolonging the half-life of the drug, reducing dose frequency, and improving the therapeutic efficacy of the said drug. The patent discloses oil-in-water type nanoemulsion composed of 1-18% of quinapril hydrochloride, 15-40% of surfactant, 0-20% of cosurfactant, and 1-20% of rose oil along with the balanced quantity of distilled water. More supportive data for granted patents in the field of nanoemulsion for antihypertensive drugs has been summarized in Table 4.

## 4. Conclusions

Nanoemulsion has progressively become the center of research and development these days. In the pharmaceutical industry, nanoemulsion has emerged as a potential and interesting drug delivery technology for cardiovascular diseases. An extensive study has been conducted on a variety of drug delivery systems, which concludes that nanoemulsion has a promising future as a delivery system for antihypertensive drugs. In the coming years, nanoemulsion will become a mainstream practice, with all positive results for the betterment of human society and an increase in life expectancy. Nevertheless, many challenges still need to be overcome to establish their safety and efficacy by performing preclinical and clinical studies. Randomized clinical trials should be performed to gain a better understanding of the effects of various crucial parameters such as droplet size, composition, and charge on the absorption, distribution, and metabolism of the antihypertensive drugs loaded in nanoemulsion. Additionally, various advancements in nanoemulsion like multiple nanoemulsions, in situ nanoemulsion, and self-emulsifying nanoemulsion are yet to be explored for cardiovascular and other diseases.

## Figures and Tables

**Table 1:** Drugs used in the treatment of hypertension along with their characteristics.

Class	Drug	Aqueous solubility	Permeability (log *P*)	Bioavailability	Ref.
Calcium channel blockers	Verapamil	7 mg/ml	3.8	10-20%	[33]
	Felodipine	7.15 *μ*g/ml	4.36	15%	[34]
	Nisoldipine	5.7 *μ*g/ml	3.1	Less than 5%	[35]
	Nitrendipine	2 *μ*g/ml	3.59	10-20%	[36]
	Amlodipine	75.3 *μ*g/ml	2.22	64%	[37]
	Nifedipine	20 *μ*g/ml	2.20	45-56%	[38]
AT1 receptor antagonist	Valsartan	0.1 mg/ml	5.8	Less than 25%	[39]
	Candesartan	5∗10^−5^ mg/ml	6.1	40%	[40]
	Irbesartan	Less than 1 mg/ml	4.5	60-80%	[41]
	Telmisartan	0.09 mg/ml	7.7	42%	[42]
	Olmesartan	7.42 *μ*g/ml	3.97	26%	[43]
*β*-Blockers	Atenolol	1.33 mg/ml	0.16	50-60%	[44]
	Metoprolol	50 mg/ml	2.15	50%	[45]
	Acebutolol	200 mg/ml	1.53	40%	[46]
	Carvedilol	0.583 *μ*g/ml	4.1	23%	[47]
Renin inhibitor	Remikiren	0.0213 mg/ml	3.9	2.5%	[48]
	Aliskiren	122 mg/ml	2.45	2.5%	[49]

**Table 2:** Summarized lipid/oil phase, surfactant, and cosurfactant used for nanoemulsion having antihypertensive drugs.

Type of excipients	Examples
Lipid/oil phase	Soybean oil, coconut oil, seasame oil, cottonseed oil, rice bran oil, Captax 355, safflower oil, Captex 8000, Myritol 318, Witepsol, isopropyl myristate, triacetin, Capryol 90, Castor oil, Sefsol-218, rapeseed oil, olive oil, peanut oil, whale oil, shark liver oil, linseed oil, palm kernel oil, corn oil, jojoba oil, citrus seed oil, almond oil, theobroma oil, ethyl palmitate, octamethyltrisiloxane, hexamethyl disiloxane, fatty ester
Surfactants	Labrafil, Cremophor EL, Lauroglycol 90, Tween 80, Tween 60, Tween 20, Span 80, Span 60, Span 40, Span 20, sodium dodecyl sulfate, lecithin, poloxamers, Labrasol
Cosurfactant	ethanol, propylene glycol, n-butanol, isopropyl alcohol, propanolol, Carbitol, polyethylene glycol 400, Transcutol

**Table 3:** Summary of selected oral antihypertensive drug-loaded nanoemulsion.

Drug candidate	Oil phase/surfactant/cosurfactant	Method of preparation	Outcomes	Ref.
Red ginger	Coconut oil/Tween 80/PEG 400	Water titration method	Red ginger provides antihypertensive action by inhibiting ACE	[88]
Nisoldipine	Peceol/Cremophor EL/Transcutol HP	Ultrasonication technique	Improved bioavailability and antihypertensive activity	[89]
Nitrendipine	Capmul MCM, Triacetin/Kolliphor ELP/Transcutol HP	Spontaneous emulsification method	Improvement in penetration of drug	[90]
Raspberry ketone	Sefsol 218®/Tween 80/Lauroglycol 90	High energy emulsification technique	Improvement in aqueous solubility and bioavailability	[91]
Eplerenone	Triacetin/Kolliphor EL/PEG 400	Ultrasonication technique	Improved bioavailability of the drug	[92]
Mebudipine	Ethyl oleate/Tween 80/PEG 400	Sonication	Improved bioavailability	[93]
Olmesartan medoxomil	Soyabean oil 700/Sefsol 218/Solutol HS 15	Phase inversion technique	Improved pharmacokinetics and therapeutic efficacy of the drug	[94]
Ramipril	Sefsol 218/Tween 80/Carbitol		The improved bioavailability of the drug	[95]
Candesartan cilexetil	Soyabean oil/Solutol HS-15/Tween 80	Solvent evaporation technique	Improved oral absorption of the drug	[96]
Valsartan	Capmul MCM/Labrafil M 2125/Tween 80		The improved oral bioavailability of the drug	[97]
Amlodipine besilate	Labrafil M/Tween 80/ethanol	Spontaneous emulsification	Relative bioavailability of was 475% than drug suspension	[98]
Telmisartan	Oleic acid/Tween 80/PEG 200	Ultrasonication	Enhanced bioavailability of the drug	[99]
Carvedilol	Peppermint oil/Tween 80/ethanol	Aqueous phase titration	Enhancement in aqueous solubility leading to improved bioavailability	[100]
Metoprolol	Isopropyl myristate/lecithin/isopropyl alcohol		Enhanced permeation of the drug through rat skin	[101]
Talinolol	Triacetin/Brij-721/ethanol	Sonication	Significant improvement in drug release, permeability, and bioavailability	[102]

**Table 4:** Summarized patent approval for nanoemulsion for antihypertensive drug.

Patent number	Therapeutic moiety	Title	Inventor	Outcomes
CN105997873A	Terazosin	Oil-in-water type terazosin nanoemulsion antihypertensive drug	Zhang Hongli	The prolonged half-life of the drug, reduced dose frequency, and improved therapeutic efficacy
CN106137958A	Apigenin	A kind of compound apigenin nanoemulsion antihypertensive drug	Zhang Hongli	Improved dissolution and penetration power of drug along with an increased instability
CN106176997A	Atenolol	A kind of compound atenolol nanoemulsion antihypertensive drug	Zhang Hongli	Improved dissolution and penetration power of drug along with an increased instability
CN102698245A	Quinapril	Antihypertensive drug of quinapril hydrochloride and rose oil nanoemulsion	Ouyang Wuqing, Sun Jianhong, Zhang Xiaohua	The prolonged half-life of the drug, reduced dose frequency, and improved therapeutic efficacy
CN102697900A	Spirolactone	Compound spirolactone nanoemulsion drug	Ouyang Wuqing, Sun Jianhong, Cao Tong	Improved dissolution and penetration power of drug along with an increased instability
CN106137961A	Celiprolol	A kind of oil-in-water type celiprolol nanoemulsion antihypertensive drug	Zhang Hongli	Improved stability, prolonged half-life, and enhancement in the therapeutic efficacy of the drug
CN106109410A	Hydralazine	A kind of hydralazine nanoemulsion antihypertensive drug	Zhang Hongli	The prolonged half-life of the drug, reduced dose frequency, and improved therapeutic efficacy
CN105997874A	Sotalol	Oil-in-water type sotalol nanoemulsion antihypertensive drug	Zhang Hongli	The prolonged half-life of the drug, reduced dose frequency, and improved therapeutic efficacy
CN102716158A	Mecamylamine	In-water type mecamylamine and celery seed oil nanoemulsion antihypertensive medicine	Ouyang Wuqing, Sun Jianhong, Gao Qing	Improved antihypertensive activity, the prolonged half-life of the drug along with reduced dose frequency
CN106177511A	Alprenolol	A kind of compound alprenolol nanoemulsion antihypertensive drug	Zhang Hongli	Improved antihypertensive activity, the prolonged half-life of the drug along with reduced dose frequency
